# PET-Scan Shows Peripherally Increased Neurokinin 1 Receptor Availability in Chronic Tennis Elbow: Visualizing Neurogenic Inflammation?

**DOI:** 10.1371/journal.pone.0075859

**Published:** 2013-10-14

**Authors:** Magnus Peterson, Kurt Svärdsudd, Lieuwe Appel, Henry Engler, Mikko Aarnio, Torsten Gordh, Bengt Långström, Jens Sörensen

**Affiliations:** 1 Department of Public Health and Caring Sciences, Family Medicine and Clinical Epidemiology, Uppsala University, Uppsala, Sweden; 2 Uppsala PET Centre, Department of Radiology, Oncology and Radiation Sciences, Uppsala University, Uppsala, Sweden; 3 Uruguayan Centre of Molecular Imaging (CUDIM), Faculty of Medicine and Faculty of Sciences, University of the Republic, Montevideo, Uruguay; 4 Department of Surgical Sciences, Pain Research, Uppsala University, Uppsala, Sweden; 5 Department of Biochemistry and Organic Chemistry, Uppsala University, Uppsala, Sweden; 6 Neuropsychopharmacology Section, Faculty of Medicine, Imperial College, London, United Kingdom; Charité University Medicine Berlin, Germany

## Abstract

In response to pain, neurokinin 1 (NK1) receptor availability is altered in the central nervous system. The NK1 receptor and its primary agonist, substance P, also play a crucial role in peripheral tissue in response to pain, as part of neurogenic inflammation. However, little is known about alterations in NK1 receptor availability in peripheral tissue in chronic pain conditions and very few studies have been performed on human beings. Ten subjects with chronic tennis elbow were therefore examined by positron emission tomography (PET) with the NK1 specific radioligand [^11^C]GR205171 before and after treatment with graded exercise. The radioligand signal intensity was higher in the affected arm as compared with the unaffected arm, measured as differences between the arms in volume of voxels and signal intensity of this volume above a reference threshold set as 2.5 SD above mean signal intensity of the unaffected arm before treatment. In the eight subjects examined after treatment, pain ratings decreased in all subjects but signal intensity decreased in five and increased in three. In conclusion, NK1 receptors may be activated, or up-regulated in the peripheral, painful tissue of a chronic pain condition. This up-regulation does, however, have moderate correlation to pain ratings. The increased NK1 receptor availability is interpreted as part of ongoing neurogenic inflammation and may have correlation to the pathogenesis of chronic tennis elbow.

**Trial Registration:**

ClinicalTrials.gov NCT00888225 http://clinicaltrials.gov/

## Introduction

Musculoskeletal pain is a common problem with an estimated cost of 2.9% of the gross domestic product (GDP) in the US [1]. Tennis elbow (TE), i.e. pain from the common extensor tendon on the lateral epicondyle, has a prevalence of 1-3% in the population [2,3]. The etiology, pathophysiology and healing mechanisms of tendon disorders are only partly known and the cause of pain in chronic tendon disorders is mostly unknown [4,5]. 

The initial overuse causes inflammation in the affected tissue [6,7]. The acute inflammation, through the action of macrophages and mast cells, causes release of inflammatory mediators which, in turn, activate peripheral nociceptive neurons [8]. In the chronic stage (symptoms more than 3 months), inflammatory cells are essentially absent and are replaced by signs of degeneration in the tissue [5,9,10,11]. Tissue samples from this stage of disease indicate increased amounts of neural fibers and transmitters, including substance P [12,13,14,15]. Substance P was the first neuropeptide to be discovered and belongs to a group of peptides called tachykinins. It is widely distributed in the central and peripheral nervous systems. There is evidence that substance P is not only involved in the nociceptive pathway but also contributes to local neurogenic inflammation [16,17,18]. 

The primary receptor for substance P is the neurokinin 1 (NK1) receptor. An increase of NK1 receptors has been documented in chronically painful tendon tissue [13]. The NK1 receptor received considerable scientific attention a decade ago, as animal models suggested that blockage of the NK1 receptor would reduce chronic pain [19,20,21,22]. Clinical studies of NK1 blockage in humans, however, showed little effect on pain and further research was halted [23,24]. Relatively few imaging studies have investigated the NK1 system in the human central nervous system (CNS) [25] and, to our knowledge, no imaging studies of tissue outside the CNS have been performed using positron emission tomography (PET) with an NK1 receptor specific radioligand. The aim of this study was to investigate the amounts of NK1-receptors in the painful arm of subjects with unilateral chronic tennis elbow before and after treatment according to a three-month graded exercise protocol, with the unaffected arm used as control. The effect of graded exercise on pain in chronic tendon disorders has been described in several previous studies [26,27,28].

## Materials and Methods

### Study population

The study was performed in the city of Uppsala, Sweden, and was nested in a larger randomized controlled trial of graded exercise as treatment for chronic TE (lateral epicondylosis). All 150 general practitioners and 90 physiotherapists at primary health care centres within a radius of 70 kilometers of the city of Uppsala were asked for information on subjects with long-lasting TE problems. In addition, subjects with TE symptoms were invited to participate in the trial through advertisements in the main local newspaper in order to recruit a sufficiently large number of subjects.

The inclusion criteria were: age 20-75 years, symptoms of TE for more than three months, and a verified diagnosis. Exclusion criteria were any of concomitant supinator syndrome, compartment syndrome of musculus anconeus, rhizopathy, inflammatory joint disease, fibromyalgia, previous elbow surgery, treatment by injection of steroids within the previous three months, and inability to understand Swedish. At a first appointment the diagnosis was checked by pain on palpation, stretching (Mill´s test), loading and Maudsley´s middle finger test by the same physician, a general practitioner and pain specialist (MP). One hundred and seventy-three patients were evaluated, of which 53 were excluded for reasons of incorrect diagnosis, other concomitant pain diagnoses, or interfering treatment, leaving 120 subjects as the final RCT study population.

Subjects in the RCT were consecutively invited to participate in the PET study until ten had accepted. Exclusion criteria for the PET study were any of: current medication interfering with the nervous or inflammatory system, substance abuse, pregnancy, recent or planned participation in another PET study, X-ray or other significant exposure to radiation, bilateral symptoms or other pain diagnosis of the upper extremities. All subjects gave written informed consent before entering the study. The Regional Ethical Review Board in Uppsala, Sweden and the Radiation Safety Committee in Uppsala, Sweden approved the study. The trial is registered as NCT00888225 at http://clinicaltrials.gov/.

### PET examination and exercise treatment procedure

The PET examinations were performed twice in each participant, before and after the treatment protocol of the RCT. The treatment consisted of a three-month daily exercise regime performed at home, with gradually increasing load on the extensor muscles of the affected forearm. The protocol has been previously described in detail [29]. Pain was rated on a 100 mm visual analogue scale (VAS), during maximum voluntary contraction of the forearm extensor muscles.

Prior to scanning, all participants refrained from analgesics for one day and anti-inflammatory drugs for three days. They also abstained from tobacco, alcohol and caffeine for twelve hours before, and from food for three hours before PET investigations.

Examinations were performed with the NK1 specific radioligand [^11^C]GR205171, synthesized according to standard manufacturing procedures and previously published methods [30] at the chemistry section of the Uppsala PET Center. The scanning procedure was executed with a Siemens ECAT EXACT HR+ whole body tomograph (CTI, Knoxville, TN, USA). The scanner enables acquisition of 63 contiguous planes of data with 2.46 mm plane spacing resulting in a total axial field of view of 155 mm.

**Figure 1:**
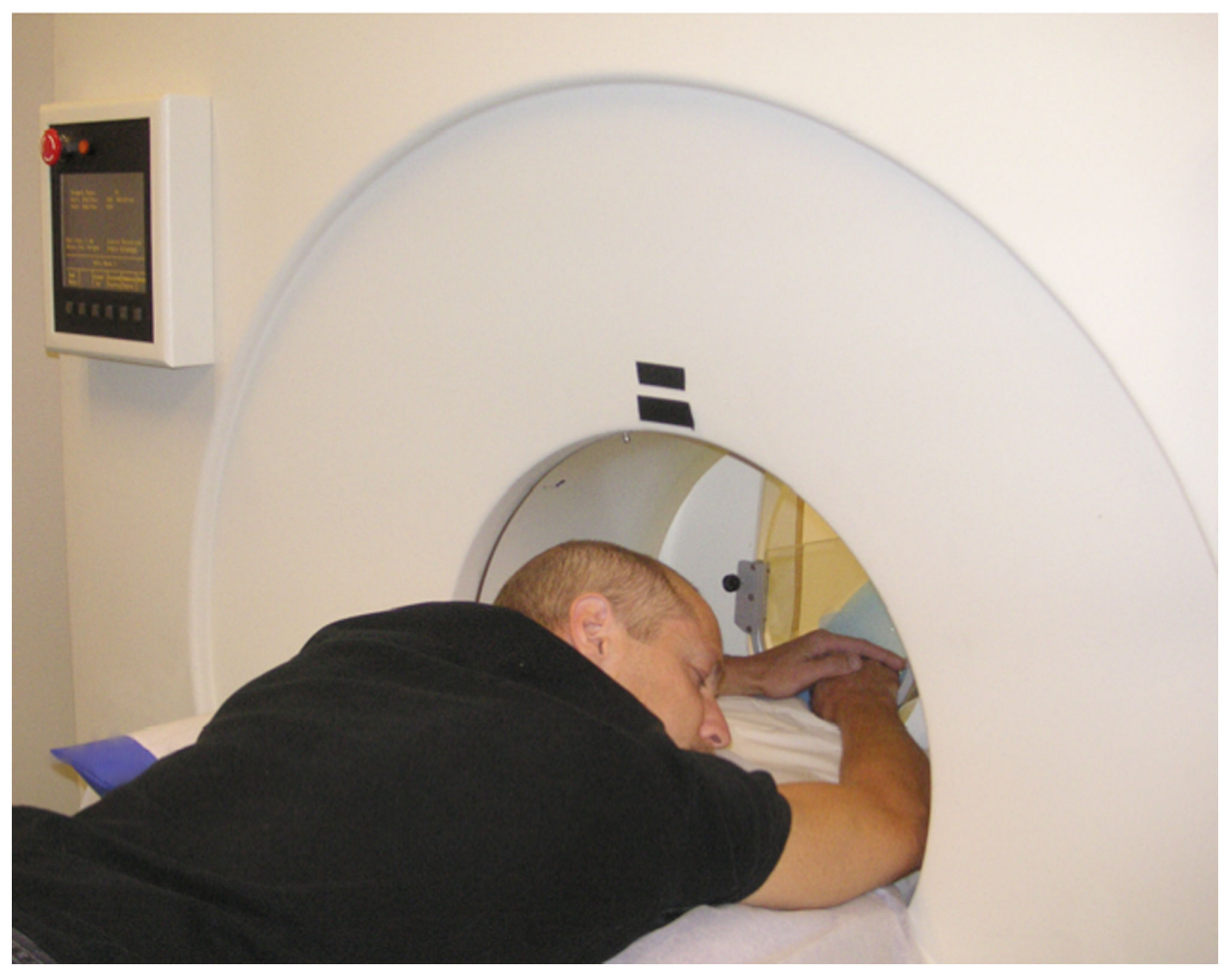
Position in the PET-scanner. The subject of the photograph has given written informed consent, as outlined in the PLOS consent form, to publication of the photograph.

Subjects were placed in prone position in the scanner with their arms stretched above the head and gently fixated, so that the elbow joints of both arms were in the field of view (Figure 1). A venous catheter was inserted in the foot and a bolus of the radioligand was injected intravenously approximately 50 minutes prior to the elbow investigation. The amount of injected radioactivity was approximately 5.6 MBq/kg bodyweight, average dose 405 (SD 17.4) MBq. A dynamic examination of other tissues was performed during the first 45 minutes. Then the imaging data was collected during a ten-minute time frame. Finally, a ten-minute transmission scan was performed using three retractable ^68^GE rotating line sources.

Emission scans were reconstructed using an ordered subset expectation maximization (OSEM) method with six iterations and eight subsets using an eight mm Hanning filter, zoom two point five. The PET data were reconstructed to a 128x128 matrix with filtered back projection and corrected for photon attenuation, decay, scattered radiation and random coincidences according to standard procedures [31].

### Data analysis

The image data were analyzed according to a non-observer dependent statistical approach. A full description including tutorial, source code and imaging data test files is available as supporting information supplement, File S3-S7. The original three-dimensional matrices representing radioactivity concentration (signal intensity) and the density maps used to correct for attenuation were loaded into ImageJ, (a public domain Java image processing program developed at the National Institutes of Health) [32,33]. An algorithm was constructed with which the left and right arms were semi-automatically located and segmented from the density maps. Based on this segmentation, the total number of voxels, their mean signal intensity, and the standard deviation (SD) of this signal intensity were calculated for each arm. The mean signal intensity of all voxels in the unaffected arm was used as reference.

Then two new image matrices were created, in which the voxel data represented the signal intensity, measured in SD units (Z score). From each Z score image, the signal intensity of voxels located >2.5 SD above reference in each arm was computed. In order to obtain a composite measure of voxel volume and signal intensity, a “Volume intensity score” was calculated by multiplying the volume of voxels with signal intensity > 2.5 SD above reference in each arm, by the summed Z score signal intensity of this volume. Two subjects discontinued exercise intervention and declined participation in the PET examination after treatment due to “lack of time”.

The derived data were analyzed using SAS software, version 9.2. Differences between the arms were computed with Student’s t-test and with unbalanced analysis of variation, both methods giving almost identical results. All statistical tests were two-tailed. P-values less than 0.05 were regarded as statistically significant. The correlation between pain rating and the Volume intensity score was calculated according to Spearman.

## Results

### Baseline characteristics of the study population

The baseline characteristics of the study population are shown in Table 1. Mean age was 49 years, five of the ten participants were women, three had a college or university education and two were current smokers. Five were office workers and five were craftsmen. Eight stated that their work consisted of manual tasks and six suspected repetitive movement as cause of their TE condition. Five had had one previous episode of TE and five had none. Mean duration of the present episode was 12 months (range 3-36). All but one had received treatment during the current episode and the majority had been treated with anti-inflammatory medication orally or by injection.

**Table 1:** Baseline characteristics of the study population.

			n	mean (SD) or %
N			10	
Age, years				48.7 (8.5)
Women, %			5	50
Smoking habits				
	Never smoked		5	50
	Ex-smokers		3	30
	Current smokers		2	20
Lateral epicondylosis history				
		Duration of present episode, weeks		52.0 (42.9)
Previous treatments given				
		NSAID	4	40
		Acupuncture	4	40
		Steroid injections	3	30
		Stretching	4	40
		Orthosis or other fixative	3	30
		Massage	1	10
		Rest	1	10
		No previous treatment	1	10

#### Neurokinin 1 receptor availability

**Figure 2:**
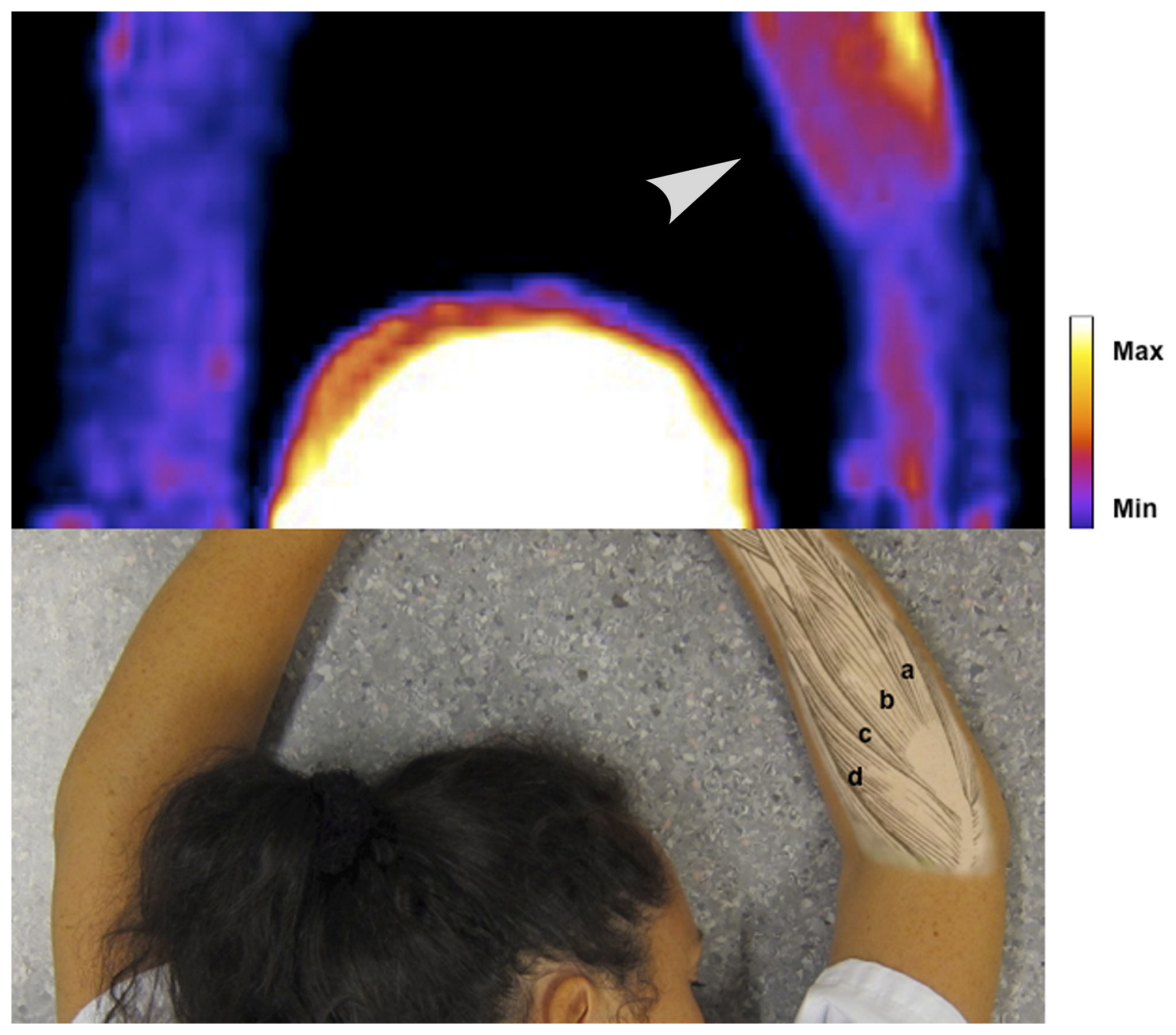
PET image of NK1 receptor radioligand [^11^C]GR205171 in subject A (out of ten subjects in total). PET images are coronal maximum intensity projections of parametric images processed as described in the methods section and in the online only supplement. Upper half of picture shows head and arms of the subject. Arrowhead indicates affected arm. Lower half of picture shows anatomical references in an unrelated subject. Forearm muscles from top to bottom: a) m. extensor carpi ulnaris, b) m extensor digitorum communis, c) m. extensor carpi radialis brevis, d) m. extensor carpi radialis longus. The subject has given written informed consent, as outlined in the PLOS consent form, to publication of the photograph.

**Figure 3:**
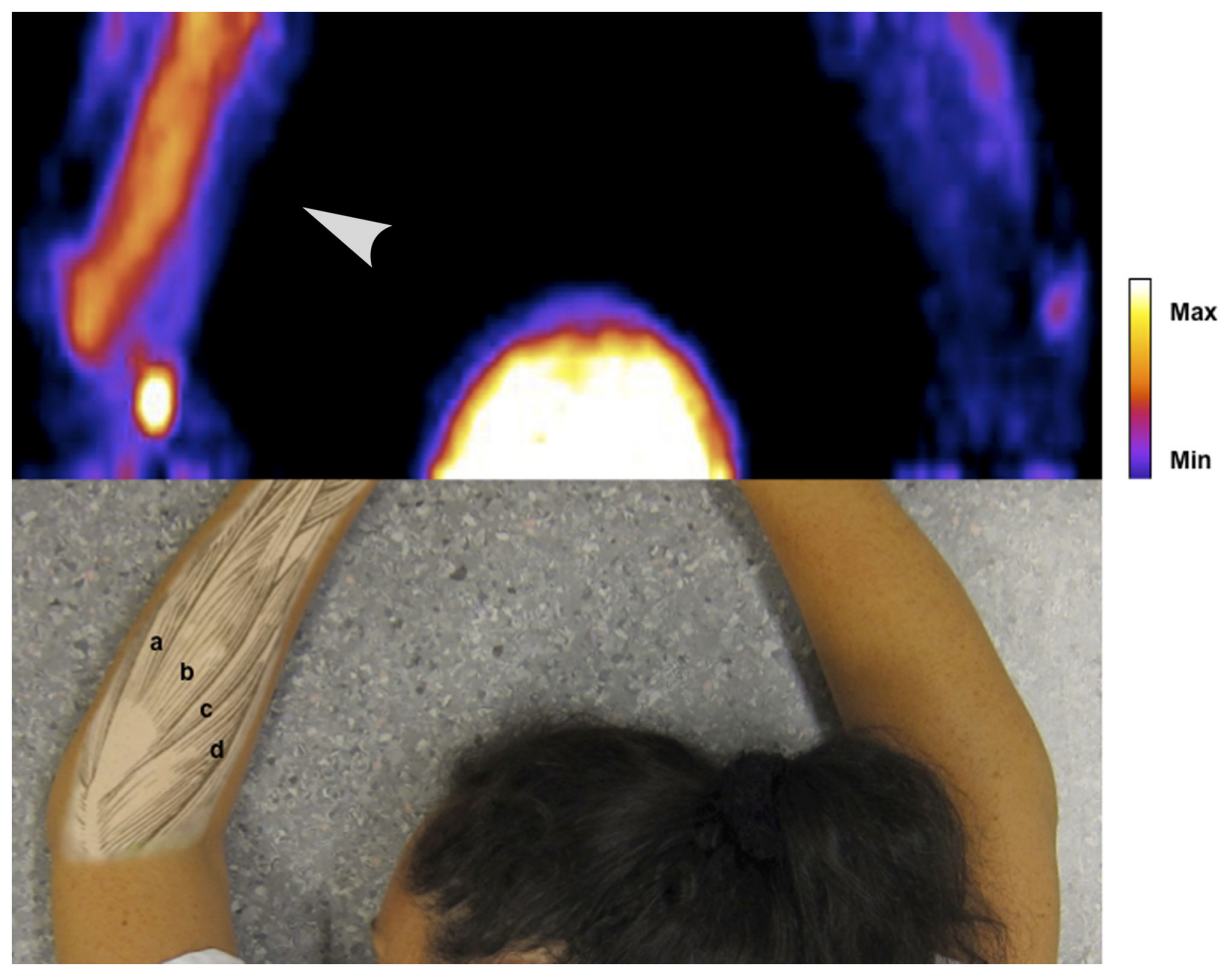
PET image of NK1 receptor radioligand [^11^C]GR205171 in subject B (out of ten subjects in total). PET images are coronal maximum intensity projections of parametric images processed as described in the methods section and in the online only supplement. Upper half of picture shows head and arms of the subject. Arrowhead indicates affected arm. Lower half of picture shows anatomical references in an unrelated subject. Forearm muscles from top to bottom: a) m. extensor carpi ulnaris, b) m extensor digitorum communis, c) m. extensor carpi radialis brevis, d) m. extensor carpi radialis longus. The subject has given written informed consent, as outlined in the PLOS consent form, to publication of the photograph.

**Figure 4:**
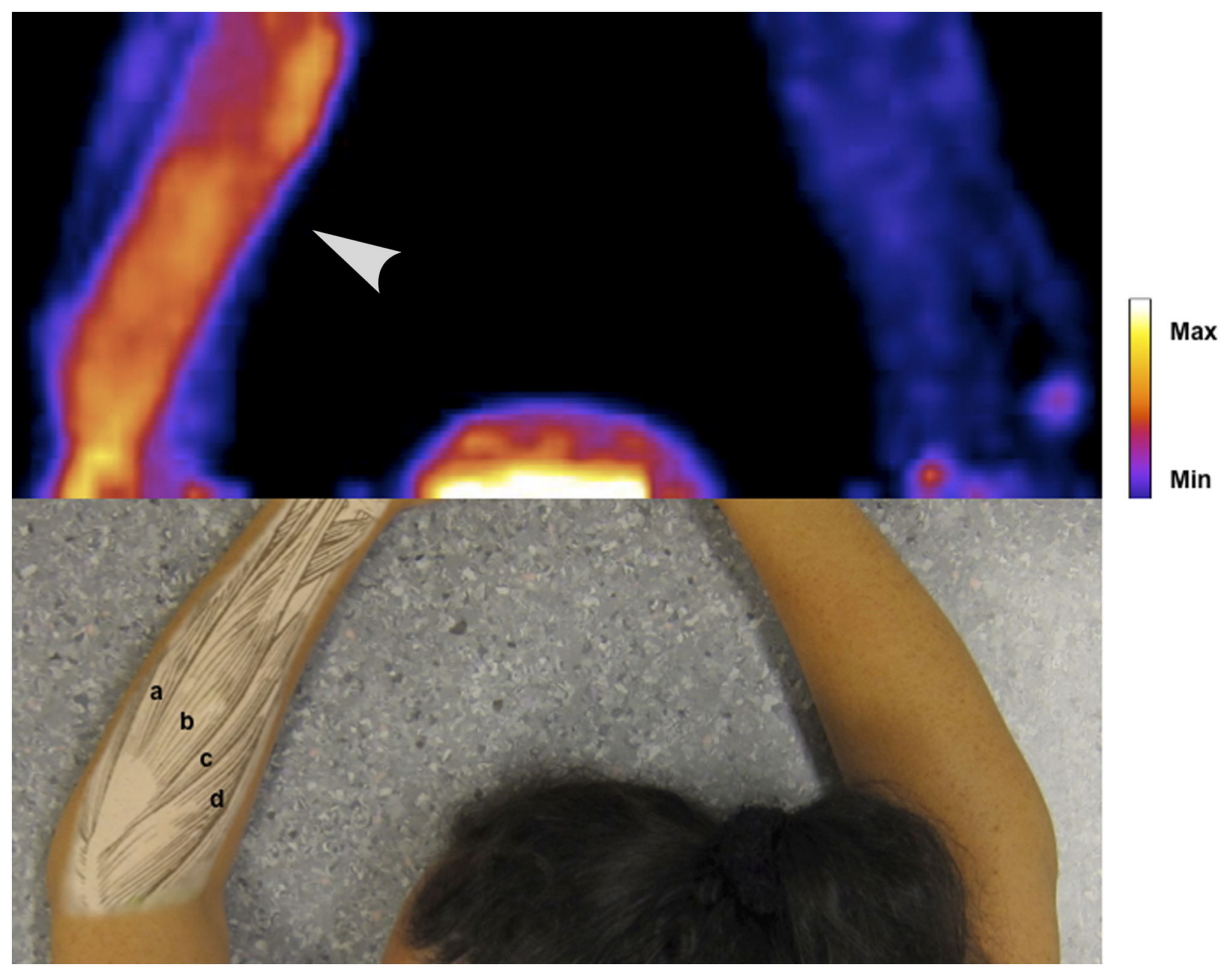
PET image of NK1 receptor radioligand [^11^C]GR205171 in subject C (out of ten subjects in total). PET images are coronal maximum intensity projections of parametric images processed as described in the methods section and in the online only supplement. Upper half of picture shows head and arms of the subject. Arrowhead indicates affected arm. Lower half of picture shows anatomical references in an unrelated subject. Forearm muscles from top to bottom: a) m. extensor carpi ulnaris, b) m extensor digitorum communis, c) m. extensor carpi radialis brevis, d) m. extensor carpi radialis longus. The subject has given written informed consent, as outlined in the PLOS consent form, to publication of the photograph.

Pain ratings and results from the analysis of the voxel data are shown in Table 2. The pain ratings were higher in the affected arm in all subjects, and decreased in all subjects after treatment. The number of voxels in the field of view was similar in both arms of each individual. The volume of voxels with signal intensity > 2.5 SD above reference was significantly higher in the affected than in the unaffected arm before treatment (150 ± 90 versus 4 ± 10 mL, p<0.001) and remained higher after treatment (212 ± 148 versus 41 ± 112 mL, p = 0.02). The mean signal intensity of this volume, measured as SD above reference, was also significantly higher in the affected than the unaffected arm before (p<0.001) and after (p=0.02). The Volume intensity score was, consequently, also significantly higher. These results are illustrated by PET image in Figure 2, 3 and 4.

**Table 2:** Data on pain ratings, and volume and intensity of the NK1 receptor radioligand [^11^C]GR205171 for the 10 subjects before and after treatment.

Subject and arm		Pain (VAS)	Examined voxels (No.)	Mean intensity (Bq)(SD)	Voxels >2.5 SD above mean of unaffected arm		
					Volume (ml)	Mean intensity (SD)	Volume intensity score (ml*SD)
1	Unaffected before	5	11675	2086 (1371)	1.7	2.9	5
1	Affected before	58	11495	2999 (1858)	110	3.7	406.5
1	Unaffected after	3	11162	1199 (668)	7	3.5	24.2
1	Affected after	28	11110	1381 (767)	30	3.3	98.5
2	Unaffected before	5	9825	4664 (3748)	0.26	2.8	0.7
2	Affected before	65	9626	5952 (4699)	30.5	3.7	112.6
2	Unaffected after	1	10776	2129 (1744)	0	0	0
2	Affected after	10	10991	2129 (1744)	172	4.4	755.4
3	Unaffected before	3	9811	1837 (804)	0	0	0
3	Affected before	67	10274	3137 (2483)	186	5.2	970.1
3	Unaffected after	4	10025	1472 (773)	0	0	0
3	Affected after	35	10299	2258 (1953)	150	4.7	702.7
4	Unaffected before	0	20009	5316 (2404)	32	3.3	106.2
4	Affected before	52	17661	5658 (2382)	31.8	3.3	104.7
4	Unaffected after	.	.	.	.	.	.
4	Affected after	.	.	.	.	.	.
5	Unaffected before	4	15012	1432 (647)	0	0	0
5	Affected before	69	15139	3174 (4802)	211	12.4	2616.5
5	Unaffected after	8	16057	2184 (3024)	0	0	0
5	Affected after	29	16753	4740 (3922)	319	4.6	1462.4
6	Unaffected before	2	16525	1956 (663)	0	0	0
6	Affected before	41	19007	2537 (1696)	196	5.4	1064.1
6	Unaffected after	5	18037	1492 (754)	0	0	0
6	Affected after	25	19571	2734 (2280)	413	4.8	1973.7
7	Unaffected before	6	12373	2953 (1844)	0.4	3.7	1.5
7	Affected before	62	15185	6573 (6433)	318	5.6	1773.6
7	Unaffected after	3	15493	2495 (1266)	0	0	0
7	Affected after	4	13906	5309 (4530)	316	5.9	1186.3
8	Unaffected before	7	13718	3056 (1118)	0.2	2.7	0.5
8	Affected before	68	15010	4501 (3109)	202	5.3	1072.2
8	Unaffected after	7	13127	2058 (766)	0	0	0
8	Affected after	67	14828	3783 (3486)	297	7.1	2120.7
9	Unaffected before	7	13512	1715 (916)	0	0	0
9	Affected before	49	13118	2023 (1466)	89	3.5	314.8
9	Unaffected after	3	13204	1966 (1133)	0	0	0
9	Affected after	2	12409	1485 (803)	0.3	2.8	0.84
10	Unaffected before	6	15999	2907 (1073)	0.4	3.3	1.3
10	Affected before	44	16734	3301 (1889)	123	4	487.8
10	Unaffected after	.	.	.	.	.	.
10	Affected after	.	.	.	.	.	.
Mean							
	Unaffected before	5	13846	2792 (1459)	3.5	1.9	11.5
	Affected before	58	14325	3985 (3082)	149.7	5.2	892.3
	p	<0.0001	0.74	0.08	<0.0001	0.003	0.003
	Unaffected after	4	13485	1874 (1266)	0.9	0.4	3.0
	Affected after	25	13733	2977 (2436)	212	4.7	1038
	p	0.01	0.9	0.06	0.001	<0.0001	0.002

**Figure 5:**
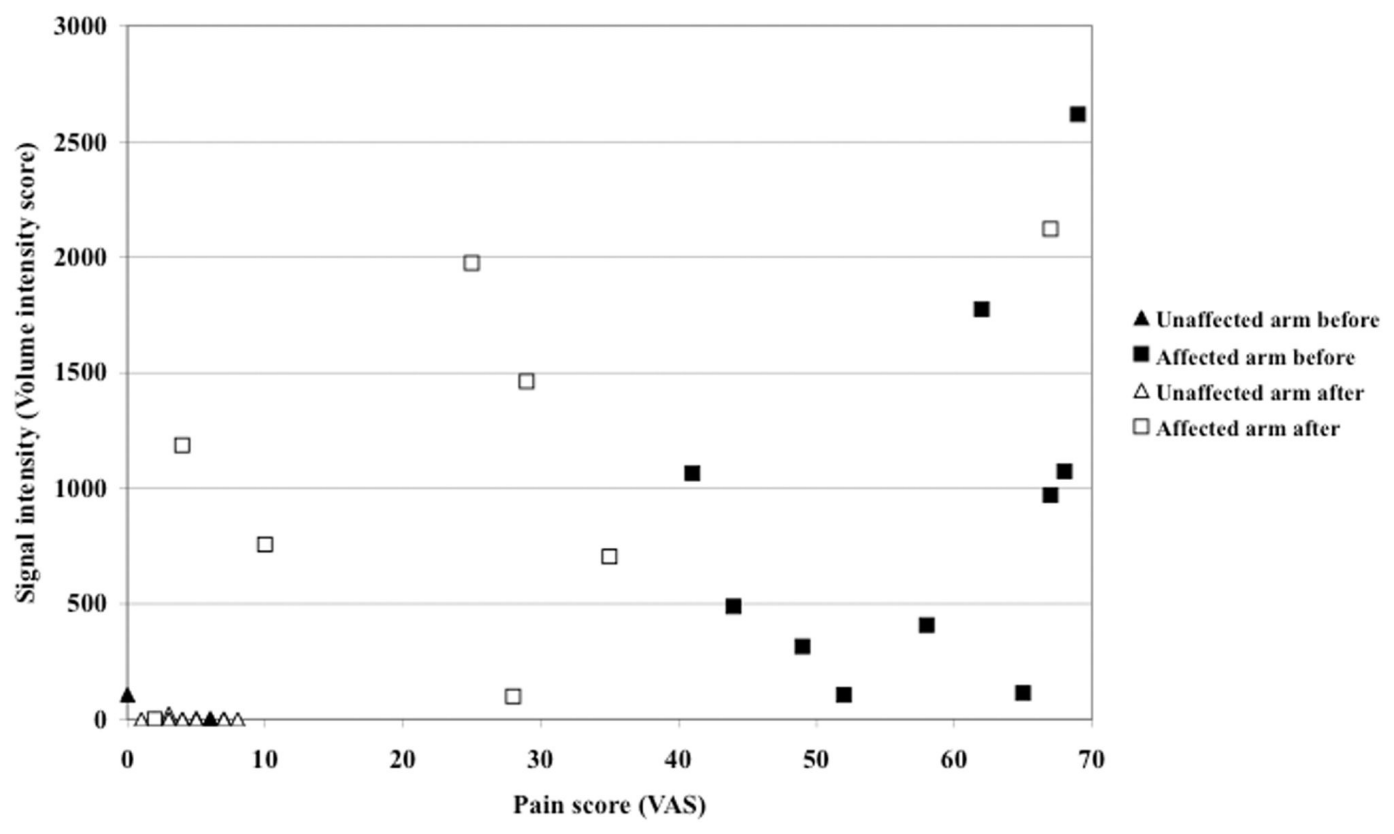
Correlation plot of pain and signal intensity. Affected and unaffected arms, before and after treatment.

In the eight subjects examined after treatment, pain ratings decreased in all subjects, but the radioligand signal intensity decreased in five and increased in three. As compared between the affected and unaffected arms, before and after treatment, pain scores and signal intensity have a strong correlation (R=0.70), but within the group affected arms, correlation is weak (R=0.30) (Figure 5). 

## Discussion

PET scan, with the NK1 specific radioligand [^11^C]GR205171, in subjects with chronic unilateral TE showed significantly higher voxel volume and signal intensity of this volume in the affected than in the unaffected arm. The correlation between pain ratings and signal intensity was strong within the group affected and unaffected arms, but weak within the group affected arms.

The radioligand [^11^C]GR205171 has, in human beings, so far only been used for studying the CNS, where it has high affinity for the NK1 receptor and displays very slow dissociation [30]. Sex and age affect NK1 receptor presentation in the CNS. It has been a matter of discussion whether [^11^C]GR205171 can be displaced by endogenous substance P [30,34]. We assume that these phenomena occur similarly in peripheral tissue and similarly in both arms of an individual.

For interpretation of PET data it is common to define a region of interest (ROI) in one or a few of the available tomography planes, where signal intensity is measured and compared to a reference region. This allows only for a limited three-dimensional evaluation of the acquired scanner data. Tennis elbow affects the extensor muscles of the forearm as well as their tendinous insertions on the lateral epicondyle. This represents an extended three dimensional tissue volume, which makes it difficult to capture by ROI analysis. In ROI analysis, the observer defines the region or volume to be compared, which also makes the method subject to observer bias. The analysis method of comparing the number, volume, and signal intensity of all voxels above a pre-set threshold of signal intensity presented here, is less subjective. This method is well suited for statistical analysis but it does not provide information on the location in the tissue. Data analysis needs to be accompanied by images to obtain this information. 

Pain ratings by VAS is a subjective measure with considerable inter-individual variability but reliable for intra-individual measurements over time [35,36].

Overuse of the muscle and tendon unit causes tissue damage that creates an acute inflammatory response [6,7]. Damaged tissue cells expose “danger-signals” that trigger macrophages and mast cells to release inflammatory mediators [8,37]. These mediators cause dilation and increased permeability of blood vessels, production of prostaglandins and leukotrienes and activation of the complement system, as well as excitation and sensitization of sensory nerves – peripheral as well as central. The sensitization of the peripheral nerves leads not only to increased excitability but also to endogenous production and subsequent release of neurotransmitters such as substance P, neurokinin A (NKA) and calcitonin gene related peptide (CGRP). Peripheral C-nociceptors can be subgrouped into peptidergic and non-peptidergic. The peptidergic nociceptors use primarily substance P and CGRP as signalling molecules, whereas the non-peptidergic nociceptors primarily use glutamate. Most of the substance P will be released by the peripheral end of the peptidergic nociceptors, where it stimulates the inflammatory cascade [38,39].

The primary receptor for substance P is the NK1-receptor. It is widely distributed in the central nervous system but has also been identified on or within immunologic cells, fibroblasts, tenocytes, endothelial cells, synovial cells, keratinocytes and osteoclasts [13,17,40]. “New” substance P-like peptides (Hemokinin 1, Endokinin A/B) have been identified in non-neural cells from immune, endothelial and placenta tissue [41]. They, too, seem to act on the NK1 receptor, which makes the cellular interaction even more intricate. NK1-receptor mRNA increases significantly, in the dorsal horn as well as in peripheral tissue, in response to peripherally induced inflammation [42]. 

The PET scans in this study revealed a high degree of unilateral and localized allocation of the radioligand [^11^C]GR205171. The specificity of [^11^C]GR205171 on NK1 receptors has been documented [30]. Locally increased blood flow, as well as NK1-receptors on endothelial cells of capillaries could explain some of the allocation. The difference in signal intensity between the affected and unaffected arms is however substantial and it is unlikely that blood flow is the single explanation. Increased amounts of substance P and NK1 receptors have been documented in histological samples of chronically painful tendon tissue [12,13]. The focal allocation of [^11^C]GR205171 is therefore interpreted as, at least partly, due to locally increased presentation of NK1 receptors.

 There is no evidence of NK1 receptors on peripheral nerve cells but the allocation of [^11^C]GR205171 may represent NK1 expression on non-neural cells such as immune and tissue cells. Immune cells known to express NK1 receptors are macrophages, mast cells and lymphocytes. Tissue cells known to express NK1 receptors are tenocytes, fibroblasts, endothelial cells and synovial cells [13,17].

Increased expression of NK1 receptors is known to occur as part of acute inflammation [42] and has been documented in chronically painful tendon tissue [13]. To our knowledge, however, this is the first time increased expression of NK1 receptors in peripheral tissue has been visualized by PET in a chronic pain condition. The reason for pain in such a condition is still uncertain. Central sensitization is well documented and most likely part of the cause. Based on the findings of this and other studies it seems likely that there is also peripheral pathology. This pathology may at least partly be explained by chronic neurogenic inflammation consisting of tripartite interaction among the immune cells, tissue cells and nerves. It is different from acute inflammation, which is dominated by inflammatory cells, local edema and increased blood flow [5,43], but shares the feature of pain. The endogenous production and release of substance P and other neurotransmitters from peripheral nociceptive neurons creates the prerequisites for a vicious circle where mastcells and macrophages could be stimulated by substance P to release algogenic substances. This may at least partly explain the longevity of the condition. There is also evidence that substance P and NK1 receptors are involved in cell proliferation and tissue remodelling [44]. This suggests the substance P-NK1 system may be involved in cell-to-cell signalling other than, or indirectly to, pain signalling.

Despite promising results in rodents, systemic blockade of NK1 receptors in human beings has not shown any convincing analgesic effect [24]. Transient presentation of the NK1 receptor has been suggested as one possible explanation, and this is supported by evidence that the NK1 receptor can be internalized [45,46]. Overlapping pathways for signal transduction may be another explanation of why blockade of only one path does not have a significant effect [24]. In the nerve system of human beings there are several overlapping pathways for pain signalling, which seem to be part of the phylogenetic evolution of a robust sensory system. This may in part explain the better effect in rodents.

If neurogenic inflammation is part of the pathology in chronic tendon pain, systemic blockade of NK1 receptors may be effective in this and perhaps in other chronic inflammatory conditions [17,23]. Clinical studies of NK1 receptor blockers that failed to deliver analgesic effects in human beings were mostly done on models of acute pain, not inflammatory pain [23]. 

There is now an NK1-antagonist clinically available and approved for the treatment of chemotherapy-induced nausea [47]. The effect of NK1-antagonism on inflammation and inflammatory pain could, for example, be studied in cancer-patients receiving this NK1-blocker. Any available experiences on inflammation and inflammatory pain from the clinical trials on NK1-antagonism and depression would be very valuable, as well as any results suggesting effects on inflammation in the preclinical trials on animals. Randomized clinical trials on the effects of NK1-antagonism on inflammation and inflammatory pain need to be performed. 

Local treatment, as well as combination therapy aimed at NK1 receptors and other receptor systems e.g. opioid receptors, also remain to be studied [48,49]. Treatments affecting the substance P-NK1 system may, in other words, not fully have played out their role [16].

PET has an unexplored potential in research of physiological processes associated with pain, not only in the CNS but also in peripheral tissue[50]. The NK1 specific radioligand [^11^C]GR205171 can be used to study the substance P-NK1 system and may with a larger study population be used to study the NK1-receptor distribution in the CNS, and relate this to abnormalities in peripheral tissue. Other tracers may also be developed to study other receptor systems such as the glutamate–NMDA/AMPA, NGF–TrkA or CGRP–CGRP-receptor. The combination of PET and functional magnetic resonance tomography (fMRI) , now available, provides a tool for detailed anatomical mapping along with the study of physiological processes. 

In conclusion, the findings of this study suggest a role for the NK1 receptor in the peripheral tissue of a chronic, soft tissue pain condition such as chronic TE. This increased NK1 receptor availability is interpreted as part of a process that can be labelled neurogenic inflammation. More research is needed to characterize neurogenic inflammation and its relation to pain and tissue remodelling. 

## Supporting Information

File S1 **Trial protocol (Swedish).**
(DOC)

File S2 **Translation of trial protocol.**
(DOCX)

File S3 **Data analysis source code and tutorial.** Tutorial and source code for non-observer dependent statistical image data analysis.
(DOC)

File S4 **Test file emission image.**
(HDR)

File S5 **Test file emission image.**
(IMG)

File S6 **Test file transmission image.**
(HDR)

File S7 **Test file transmission image.**
(IMG)
