# Supplementary material for: PET-Scan Shows Peripherally Increased Neurokinin 1 Receptor Availability in Chronic Tennis Elbow: Visualizing Neurogenic Inflammation?
Source: PLoS One. 2013 Oct 14;8(10):e75859. doi: 10.1371/journal.pone.0075859 (PMC3796513; doi:10.1371/journal.pone.0075859)
Supplement: File S1 — Trial protocol (Swedish). (DOC) [file pone.0075859.s001.doc]

Studieprotokoll 040211

**Tennisarmbåge – etiologi, excentrisk träning och hälsoekonomi**

Magnus Peterson, leg läk; Dag Elmfeldt, med dr, adj professor;

Kurt Svärdsudd, med dr, professor och Bertil Vinnars, leg läk

Institutionen för folkhälso- och vårdvetenskap, enheten för allmänmedicin.

Uppsala universitet, Uppsala.

Astra Zeneca R&D, Clinical Science, Mölndal

Ortopedkliniken, Centrallasarettet, Västerås

Dr Magnus Peterson

Institutionen för folkhälso- och vårdvetenskap

Allmänmedicin

Uppsala Science Park

751 85 Uppsala

tel: 070-288 27 68

e-post: magnus.peterson@pubcare.uu.se

**1) Bakgrund**

I sin enklaste form kan rörelseapparaten sägas bestå av en kontraktil enhet över en böjlig led. En muskel och dess sena fäster till två skilda ben som förenas av en led. Systemet är plastiskt och anpassar sig lätt till förändrade krav. Samtidigt har det sina begränsningar. Muskelns uttröttbarhet och träningsvärken dagen efter känner vi alla. Benens och ledens hållfasthet är också relativt väl kända. Men hur känner man egentligen senans begränsningar? Eller är problemet att man inte gör det?

Tennisarmbåge (musarm) är ett välkänt begrepp som ger associationer till långdragen smärta utan att närmare förklara genesen. Omkring 2% av den vuxna befolkningen beräknas lida av detta men prevalensen varierar med ålder och når en topp på cirka 6,5% i fyrtioårsåldern (5,35). Fler kvinnor än män drabbas. I åldern 42-44 år är prevalensen cirka 9% respektive cirka 3% (5). Tennisarmbåge är med andra ord en vanlig åkomma men de hälsoekonomiska konsekvenserna är trots detta dåligt undersökta. Orsaken är ofta repetitiv belastning och om arbetet är tungt ökar risken (9, 11, 26, 36) Senan och dess infästning protesterar då med smärta som kan vara intensiv vid provokation. Om varningssignalen tas på allvar kan man ofta lägga om belastningen, vila någon månad och helt enkelt vänta ut besvären(31). Men ibland räcker inte detta utan besvären kommer tillbaka så fort aktiviteten återupptas. En ny period med sjukskrivning och vila lindrar men vid återgång till datormusen eller hammaren återkommer smärtorna.

I det tidiga stadiet (epikondylit) föreligger inflammation (6, 17, 18, 19). Vila, antiinflammatoriska tabletter (NSAID) och kortisoninjektioner är väl beprövade och verksamma åtgärder i detta stadium. Vid mer långdragna besvär (epikondylalgi) kan man emellertid inte påvisa någon egentlig inflammation utan endast degenerativa tecken (19, 25, 28). Eftersom någon bra förklaring till smärtan vid epikondylalgi inte stått att finna har behandlingen varit trevande. Ett stort antal metoder har prövats, till exempel värme, kyla, bandage, gipsning, stretching, ultraljud, laser, massage, vibration, transkutan elektrisk nervstimualtion (TENS), akupunktur och kirurgi (8, 10, 13, 22, 27, 29, 31, 33, 34, 35). Mångfalden av behandlingsprinciper är ett tecken på att ingen haft övertygande framgång. De vanligaste kirurgiska metoderna bygger på fyra olika hypoteser kring smärtans ursprung. En metod inriktar sig på att reparera misstänkta rupturer i senan. En annan att minska spänningen i muskeln/senan. En tredje att denervera/dekomprimera nerver. En fjärde att leden skulle vara engagerad (12) Den pragmatiske kirurgen använder ofta en teknik som gör en syntes av dessa olika metoder.

Att rehabilitera muskeln/senan genom stegvis ökad träning under den mest belastande aktiviteten, den excentriska arbetsfasen, föreslogs redan 1985 av Stanish et al. Man utgick från det faktum att muskeln under excentriskt arbete (när man sänker en vikt och muskeln förlängs) förmår utveckla avsevärt mera kraft än under koncentriskt (när man lyfter en vikt och muskeln förkortas) och isometriskt (när man håller en vikt stilla) arbete (30). Excentriskt arbete ger då motsvarande högre belastning på senan/senfästet. Stanish hypotes var att senan med sin degeneration har sänkt prestanda och sökte det effektivaste sättet att höja denna. Behandlingen med excentrisk träning tycktes fungera även om smärtan och senans degenerativa inslag inte har något klart samband. Man gjorde bland annat en prospektiv studie av 200 patienter med ”kronisk tendinit” och en duration på i genomsnitt 18 månader. Efter ett excentriskt träningsprogram kunde man, vid uppföljningen efter 16 månader, se att 87% av dessa hade fullständig eller avsevärd regress av smärta och fuktionshinder (32). I klinisk praxis har dock metoden inte kommit i nämnvärd användning förrän Alfredsson et al visade att excentrisk träning av hälsenan vid långdragen hälsenetendinit (hälsenetendinos) har

god effekt avseende smärtlindring och återvunnen styrka (2). Effekten överträffar såväl kirurgi som

vanlig, koncentrisk träning (2,24). Hur den excentriska träningen åstadkommer smärtlindring vet man dock ej, främst för att etiologin vid smärta från degenerativa senor/senfästen är ofullständigt känd.

Vid hälsenetendinos har man funnit ökad koncentration av sensoriska neuropeptider i senan (1,3). Vid likartade tillstånd i axeln med inflammation respektive degeneration har man

observerat ökad förekomst av sensoriska nervfibrer i senan och omkringliggande vävnad (14). Detta väcker misstanken om att inväxt av sensoriska nervfibrer skulle ligga bakom smärtan vid långdragna besvär från sena/senfäste. Vid epikondylalgi har man vid behandlingen hittills ofta utgått från att ett förhöjt spänningstillstånd skulle föreligga i muskel/sena men även här har man nu funnit förhöjda nivåer av sensoriska neuropeptider i senan (4). Detta väcker misstanken om att inväxt av sensoriska nervfibrer skulle ligga bakom smärtan vid långdragna besvär från sena/senfäste.

Epikondylalgi är alltså ett vanligt tillstånd som ofta leder till mer eller mindre långvarig sjukskrivning. Patogenesen är delvis okänd och det råder oenighet om hur det bäst skall behandlas. Det är därför viktigt att närmare studera epikondylalgi och dess hälsoekonomiska konsekvenser samt att utvärdera nya och potentiellt kostnadseffektiva behandlingsmetoder.

**2) Syfte**

Syftet med detta arbete är att på patienter med epikondylalgi:

1) Utvärdera effekten av excentrisk träning vid epikondylalgi i jämförelse med koncentrisk träning. De viktigaste effektvariablerna är; smärta och styrka vid maximal isometrisk extension av handleden samt förmåga till dagliga aktiviteter (ADL).

2) Studera eventuellt engagemang av det perifera sensoriska nervsystemet i den laterala epikondylens senor och omgivande vävnad.

3) Undersöka effekten av excentrisk träning på det perifera sensoriska nevsystemet

i jämförelse med koncentrisk träning.

Dessutom syftar arbetet till att:

4) Belysa eventuella hälsoekonomiska vinster av excentrisk träning vid epikondylalgi i jämförelse med konventionell handläggning.

**3) Träningsstudie**

Det kliniska arbetet genomförs som en randomiserad, kontrollerad studie på två centra inom primärvården i Uppsala och Linköping. Patienterna randomiseras i två grupper för att genomgå antingen excentrisk träning med successivt ökande vikt eller vanlig koncentrisk träning med successivt ökande vikt. Behandlingen ges under tre månader och utvärdering sker vid månad 1, 2 och 3. Därefter släpps behandlingen fri varefter ny utvärdering sker vid månad 6 och månad 12. En schematisk presentation av studien ges i bilaga 1.

Patienterna tillfrågas om att delta i studien via sin husläkare eller sjukgymnast. Husläkaren fortsätter vara ansvarig för sjukskrivning och andra medicinska rutiner.

**3.1) Patienter**

Tvåhundra (200) patienter i Uppland och Östergötland i åldern 20 till 75 år med lateral epikondylalgi erbjuds via sin husläkare eller sjukgymnast ingå i studien.

Diagnosen epikondylalgi verifieras med sjukhistoria och smärtprovokation genom: 1) palpation, 2) töjning med knuten hand, inåtroterad arm och flekterad handled (Mill´s test), 3) isometrisk extension (extension mot motstånd) och 4) (Maudsley´s) middle finger extension test.

Differentialdiagnostiska överväganden görs genom: 1) palpation av supinatorslitsen och supination mot motstånd (supinatorsyndrom/radialtunnelsyndrom), 2) palpation av nervrotsutträde i nacke och foramen kompressions test (rhizopati), 3) palpation av armbågsled (synovit/kapsulit) och 4) röntgen i händelse av tidigare trauma eller misstänkt synovit/kapsulit.

Inklusionskriterier:

- Anamnestiskt och diagnostiskt verifierad epikondylalgi med smärta mer än 3 månader.
- Ålder 20 till 75 år
- Informerat samtycke med skriftligt medgivande.

Exklusionkriterier:

- Oförmåga att förstå och/eller följa träningsinstruktioner
- Oförmåga att läsa och/eller fylla i självskattningsformulär
- Oförmåga att närvara vid planerad uppföljning.
- Supinatorsyndrom
- Kompartmentsyndrom i musculus anconeus
- Rhizopati i arm
- Inflammatorisk led- eller bindvävssjukdom (inkl fibromyalgi)
- Tidigare kirurgi pga epikondylalgi.

**3.2) Metod**

Besvärsregistrering

Innan behandlingen inledes och under uppföljningen dokumenteras ett antal effektvariabler. Smärta vid maximal isometrisk extension i handled samt vid pronerad utsträckt arm med en 3 kg hantel skattas med hjälp av en visuell analog skala (VAS). Styrka vid maximal dorsalextension i handled mäts med dynamometer. Besvär vid aktiviteter i dagligt liv (ADL) skattas med två olika självskattningsformulär (DASH samt Gothenburg Quality of Life) (9, 16)

Randomisering och blindning

Patienterna fördelas konsekutivt i block om fyra på de två träningsgrupperna med hjälp av en datorgenererad randomiseringslista.

Träningen och utvärdderingen av smärta och ADL kan ej ske utan att patienter och personal är medvetna om vilken behandlingsgrupp patienten tillhör men datahanteringen kommer att vara blindad.

Vävnadsproven avidentifieras och kodas inför analys, som alltså sker blint.

Positronemissionstomografi (PET) sker också under, för bildtolkaren, blindade förhållanden.

Vävnadsprov

Från tjugo patienter ur varje grupp tas ett vävnadsprov i lokalanestesi. Provet tas som tubulär stansbiopsi med 4 mm diameter från senan för musculus extensor carpi radialis brevis (ECRB) fäste på laterala epikondylen. Detta prov delas i två delar där den ena efter formalinfixering sänds för immunohistokemisk analys av protein gene product 9.5 (PGP 9.5), substans P (SP), calcitonin gene related peptide (CGRP), neuropeptid Y (NPY) och tyroxinhydroxylas (TH). Den andra fryses i –70 grader för att senare (simultant med prover från övriga patienter) genomgå koncentrationsbestämning av SP, CGRP och NPY med hjälp av radioimmunoassay(RIA).

Positronemissionstomografi (PET)

Tio patienter ur varje grupp genomgår PET av armbågen. Två olika markörer injiceras intravenöst. Den ena är 2 - metoxy - 5 - (5 - trifluorometyl -tetrazol -1- yl) - bensyl -(2S - fenyl - piperidin - 3S - yl) - amin(referensnummer: GR-20 51 71, GLD) vilket är enC-märkt selektiv markör för NK1-receptorn (märker ut receptorer för substans P). Den andra är (N-methyl-^11^C)-D-deprenyl vilket är en C-märkt markör för inflammation. Signalintensitet i både den friska och den sjuka armbågen analyseras. Halveringstiden är cirka 20 minuter och efter cirka 2 timmar beräknas strålningen ha upphört. Den totala stråldosen blir cirka 6,4 mSv vilket motsvarar cirka 3 års bakgrundsstrålning.

Behandling

Grupp 1, excentrisk träning:

Som vikt används en femliters plastdunk som fylls med en liter vatten för kvinnor respektive två liter vatten för män. Den affekterade armen placeras med underarmen i bekväm höjd på ett bord med handen fritt hängande över bordskanten. Vikten lyfts med den friska armen för att tas emot med den affekterade armens handled i maximal dorsalextension. Därefter sänks vikten långsamt till bottenläge. Proceduren upprepas sammanlagt 45 gånger i set om 15 repetitioner i tre omgångar med någon minuts paus emellan. Vikten ökas succesivt genom att dunken varje vecka fylls med ytterligare en deciliter vatten.

Grupp 2, koncentrisk träning:

Proceduren genomförs enligt ovan men vikten tas emot med den affekterade armens handled i bottenläge. Vikten höjs tills handleden nått maximal dorsalextension för att där tas emot med den friska armen.

En sjukgymnast kontrollerar efter en vecka att instruktionerna uppfattats och att träningen utförs korrekt.

Patienterna erhåller föreslagen behandling under 3 månader med smärtskattning och styrkemätning under överinseende av sjukgymnast vid månad 1 respektive månad 2. Vid månad 3 genomgår de på nytt test av smärta och styrka samt fyller återigen i självskattningsformulär (ADL). Vävnadprov tas på nytt från de 10 patienterna ur varje grupp. Samtliga patienter följs sedan passivt under sammanlagt 12 månader. Smärt- och styrketest samt självskattningsformulär (ADL) uppepas vid månad 6 samt månad 12.

Smärtlindring

Som smärtlindring under behandlingen rekommenderas paracetamol 500mg två tabletter fyra gånger dagligen vid behov. Eventuell konsumtion registreras.

Effektvariabler

Vävnadsprov:

Vid immunohistokemi identifieras och räknas antalet nervfibrer efter märkning för GAP 43, PGP 9.5, CGRP, substans P, NPY och VIP. Vid RIA görs kvantitativ analys av substans P och CGRP mätt i fmol/ mg protein.

PET:

Signalintensitet för NK1-liganden (GR 20 51 71, GLD) och D-deprenyl.

Träning:

Som huvudeffektvariabel används smärtskattning med 100 mm VAS. Övriga effektvariabler är styrka vid dorsalextension i handled mätt i Nm samt två självskattningsformulär med lickertskala.

Registrering av data

Alla patientdata registreras på speciella formulär (bilaga 2) och överförs för vidare bearbetning i dator.

4) Undersökning av friska kontrollpersoner

Vävnadsprov:

20 friska kontrollpersoner utan sjukhistoria för armbågsbesvär rekryteras bland medicine studenter.

Från dessa tas ett vävnadsprov från armbågens senfäste för analys enligt metodbeskrivning ovan. Resultaten från detta kommer att användas som normalreferens vid analys av vävnadsprov från patienterna med epikondylalgi.

PET:

10 friska kontrollpersoner utan sjukhistoria för armbågsbesvär rekryteras bland medicine studenter Dessa genomgår positronemissionstomografi (PET) enligt metodbeskrivning ovan. Resultaten från detta kommer att användas som normalreferens vid PET av patienterna med epikondylalgi.

5) Statistisk metod

Variansanalys för kontinuerliga respektive logistisk regression för diskreta utfallsvariabler.

6) Resultatredovisning

Resultaten kommer att redovisas i form av en vetenskaplig rapport samt publikationer i olika vetenskapliga skrifter.

7) Etiska aspekter

Deltagande i studien är helt frivilligt. Patienten kan när som helst avbryta sitt deltagande utan att detta negativt påverkar hans/hennes allmänna omhändertagande. Rekryteringen inleds först efter att studien godkänts av forskningsetikkomittéerna vid universiteten i Uppsala och Linköping. Både patienterna och kontrollpersonerna erhåller såväl muntlig som skriftlig information om studien och dess olika moment (bilaga 3 och 4). Endast de patienter och kontrollpersoner som därefter ger sitt skriftliga samtycke kommer att inkluderas i studien.

Ordinarie husläkare fortsätter vara patientansvarig läkare (PAL) med ansvar för sjukskrivning och övriga medicinska spörsmål. Såväl PAL som patient kan när som helst under studien få kontakt med ansvariga studieledare (Magnus Peterson; Uppsala eller Lars Borgquist; Linköping).

8) Tidsplan

Patientinsamlande startar hösten 2003 och pågår under ett år. Insamlingstiden kan komma att förlängas om ett tillräckligt antal patienter inte har hunnit rekryteras fram till hösten 2004. Immunohistokemisk analys av vävnadsproverna görs fortlöpande under studien medan vävnadsproverna för RIA samlas in och analyseras vid ett och samma tillfälle under hösten 2004. Patienterna följs i sammanlagt ett år med uppföljning av sista patienten under hösten 2005.
