# Supplementary material for: PET-Scan Shows Peripherally Increased Neurokinin 1 Receptor Availability in Chronic Tennis Elbow: Visualizing Neurogenic Inflammation?
Source: PLoS One. 2013 Oct 14;8(10):e75859. doi: 10.1371/journal.pone.0075859 (PMC3796513; doi:10.1371/journal.pone.0075859)
Supplement: File S2 — Translation of trial protocol. (DOCX) [file pone.0075859.s002.docx]

Translation of trial protocol

Kurt Svärdsudd/Magnus Peterson

Department of Public Health and Caring Sciences

Section of Family Medicine and Clinical Epidemiology

Uppsala University, Sweden

**Assessment of the involvement of peripheral nerves in chronic epicondylitis using positron-emission-tomography (PET) and histochemical analyses**

**Background**

Epicondylitis is a common disorder which is difficult to treat, and causes disability in patients and large costs for society. The acute phase is characterized by inflammation in the tendons and their insertions on the lateral epicondylus (5, 7, 8, 9), and anti-inflammatory drugs are adequate and effective.

In chronic epicondylitis (symptoms for more than 3 months) inflammation is usually absent while degeneration predominates (9, 11, 12). The cause of pain is mainly unclear. Recent research on chronic epicondylitis, and particularly on similar disorders in tendons of the shoulder and the heel, suggests ingrowth of peripheral nerves. Both nerve fibers and neuro-peptides have been found in the affected tissue (1, 3, 4, 6). It is therefore of no surprise that anti-inflammatory drugs have little to offer in this stage. Unfortunately other treatments with proven effect are also lacking, although many have been suggested. Promising results have, however, recently been reported from training programs with increasing load on tendons and their insertions during the eccentric phase (lengthening) of muscle contraction (2, 10, 13).

This is a randomized clinical trial in patients with chronic epicondylitis to investigate the involvement of the peripheral nervous system and to evaluate the effects of eccentric exercise in comparison with concentric exercise (muscle shortening). This trial provides a unique opportunity to investigate the role of peripheral nerves in chronic epicondylitis, and the possible influence of two different training methods. The involvement of peripheral nerves in the disease process can be examined using positron-emission-tomography (PET) or histochemical analyses of biopsies. If both methods are used the information provided can be compared. The role of peripheral nerves will be studied in a subsample of patients. Healthy volunteers will be examined accordingly.

Approvals from the Regional Ethics Committee in Uppsala and the Radiation Safety Committee in Uppsala have been obtained for for both tracers GLD and D-Deprenyl.

Informed consent will be obtained from all patients and healthy volunteers. Patient data registration has been approved according to the present law (”Personuppgiftslagen”).

**Main trial**

Aim:

To evaluate the effect of eccentric excercise in comparison with concentric excercise on pain, strength and activities of daily life (ADL) in patients with chronic epicondylitis.

Methods:

Two hundred (200) patients with chronic epicondylitis will be randomized to either eccentric or concentric excercise for three months. Pain and strength will be registered before start of treatment and after 1,2,3,6 and 12 months. Activities of daily life and well-being will be examined using questionnaires (DASH and Gothenburg Quality of Life) before start of treatment and after 3,6 and 12 months. Patient data will be registered in specially designed case record forms. Results will be analyzed using analysis of variance and logistic regression.

Inclusion Criteria:

- Verified tennis elbow for more than three months
- Age 20-75 years
- Informed consent

Exclusion Criteria:

- Inability to understand or follow instructions
- Inability to read or fill in questionnaires
- Inability to participate at measurement sessions
- Supinator syndrome
- Compartment syndrome of anconeus muscle
- Rhizopathy of arm
- Inflammatory joint or soft tissue disease including fibromyalgia
- Previous surgery because of tennis elbow

Randomization and blinding

Patients are consecutively distributed in blocks of four to the two exercise groups by the aid of a computergenerated randomizationlist.

Training and evaluation of pain and ADL cannot be blinded but all handling of data will be blinded.

Tissue samples will be ananymous and coded during analysis.

Analysis of positron emission tomography (PET) images will be blinded to the analyzer.

**Substudy on peripheral nerve involvement**

Aims:

1) To assess the involvement of peripheral nerves in the tendons of the lateral epicondylus in patients with chronic epicondylitis using PET and histochemical analyses of biopsies.

2) To evaluate and compare the effect of eccentric and concentric excercise on peripheral nerves in the tendons of the lateral epicondylus in patients with chronic epicondylitis, using PET and histochemical analyses of biopsies.

Methods:

Ten patients from each treatment group (twenty in total) and ten healthy volunteers will be examined for:

1) Substance P-receptors using PET after intravenous injection of a C-marked selective NK-receptor antagonist 2 - metoxy - 5 - (5 - trifluorometyl -tetrazol -1- yl) - bensyl -(2S - fenyl - piperidin - 3S - yl) - amin (reference number GR-20 51 71, GLD).

2) Inflammation using (N-methyl-11-C)-D-deprenyl, DDE.

The patients will be examined before start of treatment and after three months. The healthy volunteers will only be examined once.

Signal intensity in both the healthy and the painful elbow will be analyzed. Half life of radioactive ligand is about 20 minutes and after approximately 2 hours the radiation will end. Total dose of radiation is 6,4 mSv equivalent to 3 years background radiation.

From 20 patients from each treatment group (40 in total) and 20 healthy volunteers a biopsy will be taken from the tendon at its insertion on the lateral epicondylus. This sample will be examined for nerve fibers and neuro-peptides using immuno-histochemistry and radioimmunoassay (RIA). Biopsies will be taken before start of treatment and after three months from the patients, but only once from the volunteers.

Patient data will be registered in specially designed case record forms. Results will be analysed using analysis of variance and logistic regression.

**Importance of the study**

Chronic pain from tendons and their insertions is a common problem that causes disability in patients and great costs for society. Lack of understanding of the mechanisms behind the pain can partly explain the fact that effective treaments are missing. It is conceivable that better understanding of the pathology in these conditions can contribute to improved treatments.

This study provides the opportunity to further evaluate the possible involvement of the periheral nervous system in the pain process in chronic epicondylits. It will also allow the investigation of the efficiency and mechanisms of different types of training.

**References**

1, Alfredson H. Glutamate NMDAR1 receptors localised to nerves in human achilles tendons. Implications for treatment? Knee Surg, Sports Traumatol, Arthrosc 2001;9:123-6

2, Alfredson H. Heavy load eccentric calf muscle training for the treatment of chronic achilles tendinosis. Am J Sports Med 1998;3:360-6

3, Alfredson H. In situ microdialysis in tendon tissue: high levels of glutamate, but not prostaglandin E2 in chronic achilles tendon pain. Knee Surg, Sports Traumatol, Arthrosc 1999;7:378-81

4, Alfredson H. In vivo investigation of ECRB tendons with microdialysis technique – no signs of inflammation but high amounts of glutamate in tennis elbow. Acta Orthop Scand 2000;71(5):475-9

5, Archambault JM. Exercise loading of tendons and the development of overuse injuries. Sports Med 1995;2:77-89

6, Hoe-Hansen C. Acute local inflammation elicits sprouting of sensory axons in the rat supraspinatus tendon. (Thesis. Feb 16, 2001. Linkoping University Medical Dissertations No. 658)

7, Kannus P. Tendons – a source of major concern in competitive and recreational athletes. Scand J Med Sci Sports 1997;7:53-4

8, Langberg H. Metabolism and inflammatory mediators in the periendinous space measured by microdialysis during intermittent isometric exercise in humans. J Physiol 1999;515:919-27

9, Leadbetter W. Cell-matrix response in tendon injury. Clin Sports Med 1992;3:533-78

10, Mafi N. Superior short-term results with eccentric calf muscle training compared to concentric training in a randomized prospective multicenter study on patients with chronic achilles tendinosis. Knee Surg, Sports Traumatol, Arthrosc 2001;9:42-7

11, Nirschl R. Elbow tendinosis/tennis elbow. Clin Sports Med 1992;4:851-70

12, Regan W. Microscopic histopathology of chronic refractory lateral epicondylitis. Am J Sports Med 1992;20:746-9

13, Stanish W.D. Eccentric exercise in chronic tendinitis. Clin Orthop Rel Res. 1986;208:65-8
