# Supplementary material for: PET-Scan Shows Peripherally Increased Neurokinin 1 Receptor Availability in Chronic Tennis Elbow: Visualizing Neurogenic Inflammation?
Source: PLoS One. 2013 Oct 14;8(10):e75859. doi: 10.1371/journal.pone.0075859 (PMC3796513; doi:10.1371/journal.pone.0075859)
Supplement: File S3 — Data analysis source code and tutorial. Tutorial and source code for non-observer dependent statistical image data analysis. (DOC) [file pone.0075859.s003.doc]

Appendix 1.

Article: PET-scan shows peripherally increased neurokinin 1 receptor availability in chronic tennis elbow: Visualizing neurogenic inflammation?

This appendix provides a detailed description of a novel method for parametric image formation and non-biased statistical analysis of such image. The method should potentially be applicable to other organs than elbows with modifications.

A software was written in the macro script language provided in the image analysis software ImageJ version 1.39J . All newer versions of ImageJ should run the macros without changes. The macros are included below and are commented to the extent, that a person with some knowledge of programming in ImageJ should be able to use and modify the code.

The idea is to find the contours of each arm in each slice of a PET transmission scan centered over the elbows of subject in the prone position in the scanner. The contours are then used to identify and measure the radioactivity from each voxel in one of the arms, and then calculate the mean and standard deviation of this activity. After that, each voxel pertaining to the other arm is analyzed and categorized as normal or abnormal, depending on a user-defined cut-off for the number of standard deviations required. In this process, a number of measurements are performed:

- The full volume of each arm within the field of view of the scanner.
- The mean radioactivity (in Bq/cc) and the standard deviation (also in Bq/cc) within each full volume.
- The volume of “abnormal” voxels in an arm, in which the radioactivity is higher than the mean + (SD*user-defined-cut-off) in the other arm.
- The fractional volume of abnormal tissue (volume of abnormal voxels within arm/full volume of arm)
- A “severity”-score, where the sum of SDs in the abnormal volume is multiplied with the abnormal volume (unit of SD*ml).

Finally, a parametric image is then created, where the radioactivity in each voxel of the entire image stack is compared to the mean activity in a defined arm, and expressed as standard deviations from this mean.

In its current form, the automated method is only applicable to PET data consisting of pairs of transmission images acquired using rotating 68-Ge/Ga rods and attenuation-corrected emission images. The current software also requires the organs (elbows) be centered within the field of view of the scanner. In this study, the Siemens/CTI ECAT HR plus scanner was used, permitting a field of view of 15.5 cm. Further, ImageJ does not internally read ECAT7 files. The original PET data files were therefore converted to the Analyze format using a commercial software package PMOD 2.8 (PMOD Technologies Ltd, Switzerland).

References

1. Abràmoff MD MP, Ram SJ (2004) Image processing with ImageJ. Biophotonics international. pp. 36-43.

Table 1. A step-by-step explanation to the inner workings of the proposed algorithm. Interested readers are referred to the source code included at the end of this appendix.

| Step | Description | |
| --- | --- | --- |
| 1 | Load Macro 1 into ImageJ and run macro. This will open a transmission image stack (Tx) and a emission image stack (Em). Tx is then converted to a binary image with pixel values of 0 or 255, using an in-built tresholding algorithm. For Tx images acquired using the HR+ scanner, the “auto treshold” setting produces reliable results. | |
|  | 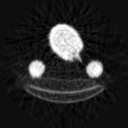  Figur 1: Original Tx | 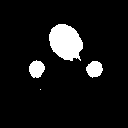  Figur 2:Binary Tx after thresholding |
| 2 | The user is requested to create a “point” ROI in both arms, starting with the arm that will be identified as “healthy”. Points are defined in the first slice of the image stack. These points needs to be added to ImageJ’s ROI manager manually.  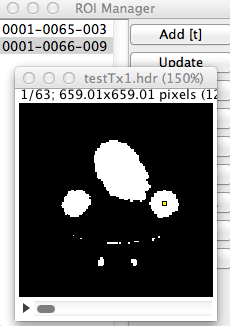 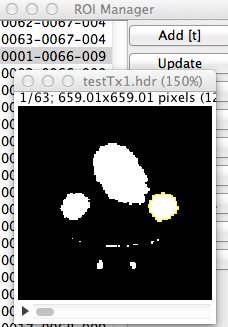  Figur 3: Placement of point ROI within segmented arm (left) with resulting automatically generated ROI (right) | |
| 3 | Load Macro 2 into ImageJ and run macro. This will open a dialog box requiring the user to identify the Em and Tx images by their titles. Further, the user can set the cut-off for the number of standard deviations (SDs) above the mean activity in the “healthy” required for a voxel in the second arm to be identified as “sick” (predefined at 2.5 SDs). | |
| 4 | Macro 2 reads the point coordinate of the “healthy” arm point ROI and finds the first pixel to the left that is not 255. This enables the “wand” tool of ImageJ to create a polygonal ROI outlining the contour of the arm within that slice. This ROI is added automatically to the ROI manager. The center of the contour ROI is calculated and used as an offset for finding the contour of the arm in the next slice. This process is then iterated until there are no more slices in the image stack. Using datasets from a HR+ scanner, there are now 63 ROIs created.  The automated ROI creation is then repeated, using the point ROI of the second arm as an entry point. | |
| 5 | The macro then goes through each ROI related to the first arm and calculates the mean and SD of the activity from the confined voxels inside ROIs. The mean is an average derived from in-built ROI measurements. The SD from all voxels is derived from a voxel-by-voxel analysis of variance, defined in the macro. Mean and SD calculations are then performed for the second arm. | |
| 6 | During the iterated voxel-wise analysis, a number of statistical parameters are calculated and printed to a log window:  Volumes of arms within the field of view.  Mean±SD of radioactivity within each arm.  Number and volume of abnormal voxels in the second arm, in which radioactivity is higher than the sum of (mean + SD*cut-off-factor). The cut-off-factor was set by the user in the initial dialog box, predefined as 2.5 SDs.  Fractional volume of abnormal voxels in the second arm (abnormal voxels/all voxels within second arm ROI contours).  Sum of “SD” values from abnormal voxels in second arm  A “sickness-severity” score is constructed by multiplying sum of SD values and volume of abnormal voxels in second arm. | |
| 7 | A final call to the subroutine SDize substitute each voxel of the entire emission image stack, where the new voxel value is calculated as:  val2 = ((val1 - mean) / sd);  where val1 is the original voxel value, mean and sd are calculated from the first arm. | |
| 8 |  | |
|  |  | |
|  | 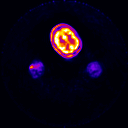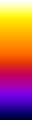  Figur 4:Transaxial section through head and elbows of original radioactivty distribution. Unit is Bq/ml | 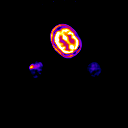  Figur 5:Same transaxial section after normalization to mean of arm in the right side of image. Unit is SD, max value set to 15 SD. |
|  |  | |
| 9 | For presentation purposes, a maximum intensity projection (MIP) of the image stack is made using in-built commands in ImageJ. | |
|  | 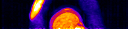  Figur 6 MIP of original distribution  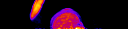  Figur 7: MIP of SD image after normalization to mean and SD of right arm. Max value in left arm is 17 SDs.  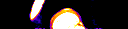  Figur 8: MIP of SD image after normalization to mean and SD of left arm. Max value in right is 0.7 SD | |
| 10 | Macro 1: Open PET images and perform thresholding of transmission image | |
|  | //May 2008-April 2013. By Jens Sörensen. Uppsala University Hospital PET Centre.  //open tendinitis images and prepare for normalization  showMessage("Open Tx then Em");  TxImage = TxOpen(); //returns last image after preparation  EmImage = EmOpen(); //same here  imageCalculator("Multiply create 32-bit stack", EmImage,TxImage);  setMinAndMax(0,6);  setTool("Point");  selectImage(TxImage);  TxTitle = getTitle();  selectWindow(TxTitle); //make masked Tx Image top  if (roiManager("count")>0) roiManager("reset");  exit();  //------------------------------------------------------------  //open Transmission image and process for analysis  //--------------------------------------------------------------  function TxOpen() {  open();  //run("Threshold...");  setAutoThreshold(); //gives approx same volumes as manual drawing  run("Convert to Mask", " black");  run("Fill Holes", "stack");  run("Divide...", "stack value=255"); // Divide all pixels by 255 to create true binary image of 0s and 1s  run("Color Balance...");  run("Enhance Contrast", "saturated=0.5"); // set color scale to 0-1  run("In");  return nImages //number of opened image is most recently created  }  //------------------------------------------------------------  //open Emission image and process  //------------------------------------------------------------  function EmOpen() {  open();  run("32-bit");  run("In");  run("Fire");  return nImages;  } | |
| 11 | Macro 2: Create ROIs and categorize voxels. | |
|  | //Feb 2008-April 2013. Part 1. By Jens Sörensen, Uppsala University Hospital PET Centre.  //Tx arms analysis. Preprocessing by threshold and "Fill holes". Divide image by 255 to  // create a mask of 0 and 1's. Add 2 ROI points to first slice of stack,  //first point in the healthy arm, second in the sick arm  requires("1.39j");  //needs dialog to identify images and set SD cut off  ImTitle = newArray(nImages); //is used to keep names of open Images  for (i=1; i<=nImages; i++) {  selectImage(i);  ImTitle[i-1] =getTitle();  }  Dialog.create("Tendinitis analysis");  Dialog.addMessage("Select name of window with emission data and Tx mask");    Dialog.addChoice("Emission data:", ImTitle);  Dialog.addChoice("Tx Mask data:", ImTitle);  Dialog.addNumber("Std is significant above:", 2.5);  Dialog.show();  petWin = Dialog.getChoice();  maskWin = Dialog.getChoice();  signifSD = Dialog.getNumber();  print("");  print("Tendinitis analysis - 11C-GLD PET");  print(petWin , maskWin);  print("Pixels included in sick volume if SD >:", signifSD);  //make sure Tx image is on top has a point ROI in either arm. These ROIs have to be defined  // in the first slice and added to the ROImanager. Then do preprocessing  setBatchMode(true);  selectWindow(maskWin);  if(roiManager("count")!=2 ) {exit()} //only 2 points defined  var x1=0;var y1=0;var x2=0;var y2=0;var z=0;var a=newArray(2);  var hArmRoiCount = 0; var sArmRoiCount = 0;  var sArm_VolScore; //used to get data from SD analysis function calcVolSD  var sArm_SDScore;  roiManager("select", 0); //healthy arm  getSelectionBounds(x1,y1,wid,hei); //wid, hei are dummies here  roiManager("select", 1); //sick arm  getSelectionBounds(x2,y2,wid,hei);  roiManager("Reset"); //clear the 2 points  //documentation  if(x1<x2) {  print("Analyzing left arm as sick.");  } else {  print("Analyzing right arm as sick");  }    //starting with healthy arm  createArmRoiStack(x1,y1);  hArmRoiCount = roiManager("count"); // probably 63 in a ECAT volume  createArmRoiStack(x2,y2);  sArmRoiCount = roiManager("count"); // rois from 'hArmRoiCount' to 'sArmRoiCount-1' belong to sick arm    //080218-130427 JS part 2  //rois created in healthy arm from Tx Image and stored in RoiManager. Make Em image upper window and run  //this part calcs mean and SD for healthy arm, then converts image to parameter SD image  selectWindow(petWin);  sum_nPixels = 0; sum_mean = 0; sum_std = 0;  n = hArmRoiCount;  for (i=0; i<n; i++) {  roiManager("select", i);  getRawStatistics(nPixel, mean, min,max,std);    sum_nPixels = sum_nPixels + nPixel;  sum_mean = sum_mean + (nPixel*mean);  sum_std = sum_std + (nPixel*std);  }  sum_mean = sum_mean/sum_nPixels;  sum_std = sum_std/sum_nPixels;  sum_nPixels = sum_nPixels/i;  setResult("nPixel", 0, sum_nPixels);  setResult("Mean", 0, sum_mean);  setResult("std", 0, sum_std);  updateResults();  //now we know the mean and we want to find true std. Have to calc variance  // by repeating roi analysis pixel by pixel  n = hArmRoiCount; tVarians = 0; tot_pixel = 0;  for (i=0; i<n; i++) {  roiManager("select", i);  getSelectionBounds(xc,yc, wid, hei);  for (y=yc; y<(yc+hei); y++) {  for (x = xc; x < (xc+wid); x++) {  val = getPixel(x,y); //z already set by previous roi selection  if (val > 0) { //pixel value inside Roi  tMean = tMean + val; //for mean calc  tVarians = tVarians + pow((val - sum_mean), 2);  tot_pixel = tot_pixel +1;  }  } //next x  } //next y  } //next i  hArm_std = sqrt(tVarians / (tot_pixel-1));  hArm_Mean =tMean / tot_pixel;  //debug stuff  print("hArm mean", hArm_Mean,"\n","hArmSTD:",hArm_std,"\n");  print("total pixels: ", tot_pixel, " mean pixel: ", tot_pixel/nSlices);  //now calc mean and SD for sick arm  sum2_nPixels = 0; sum2_mean = 0; sum2_std = 0;  for (i=hArmRoiCount; i<sArmRoiCount; i++) {  roiManager("select", i);  getRawStatistics(nPixel, mean, min,max,std);    sum2_nPixels = sum2_nPixels + nPixel;  sum2_mean = sum2_mean + (nPixel*mean);  sum2_std = sum2_std + (nPixel*std);  }  sum2_mean = sum2_mean/sum2_nPixels;  sum2_std = sum2_std/sum2_nPixels;  sum2_nPixels = sum2_nPixels/(sArmRoiCount-hArmRoiCount);  setResult("nPixel", 1, sum2_nPixels);  setResult("Mean", 1, sum2_mean);  setResult("std", 1, sum2_std);  updateResults();  //Calc pixelwise mean and Stdev from sick arm. Sa,e proc as for hArm previous  // resetting sum counters  n = sArmRoiCount; tMean = 0; tVarians = 0; tot_pixel = 0;  for (i=hArmRoiCount; i<n; i++) {  roiManager("select", i);  getSelectionBounds(xc,yc, wid, hei);  for (y=yc; y<(yc+hei); y++) {  for (x = xc; x < (xc+wid); x++) {  val = getPixel(x,y); //z already set by previous roi selection  if (val > 0) { //pixel value inside Roi  tMean = tMean + val; //for mean calc  tVarians = tVarians + pow((val - sum_mean), 2);  tot_pixel = tot_pixel +1;  }  } //next x  } //next y  } //next i  sArm_std = sqrt(tVarians / (tot_pixel-1));  sArm_Mean =tMean / tot_pixel;  //debug stuff  print("sArm mean", sArm_Mean,"\n","sArmSTD:",sArm_std,"\n");  print("total pixels: ", tot_pixel, " mean pixel: ", tot_pixel/nSlices);  SDize(hArm_Mean,hArm_std); //creates parametric image where intensity is SD's compared to hArm mean  calcVolSD(signifSD);  print("Sick pixels: ", sArm_VolScore, "\n");  print("Sick volume (mL): ", (calcVolume(sArm_VolScore)/1000));  print("Sick Volume (%): ", sArm_VolScore/tot_pixel*100);  print("Sickness severity (sum SD in sick pixels): ", sArm_SDScore, "\n");  //------------------------------------------------------  // this function receives the midpoint of the arm in the first slice  // it then creates a roi by moving the point x,y to the left until this point  // is outside the arm mask. Then wands the arm region and add this region to  // the ROI manager. Works thru the stack each slice. No return value  function createArmRoiStack(x,y) {  for(z=1;z<=nSlices;z++) {  setSlice(z);  do {  x--; //go left  } while (getPixel(x,y) == 255);  doWand(x,y);  roiManager("Add");  getSelectionBounds(x,y,wid, hei);  x=x+wid/2; y = y+hei/2; //new center coords, so x1,y1 is within arm in next slice  } //next z  }  //---------------------------------  function SDize(mean, sd) {  depth = nSlices; wid = getWidth(); hei = getHeight();  for(z=0;z<nSlices;z++) {  setZCoordinate(z);  for(y=0;y<hei;y++){  for(x=0;x<wid;x++) {  val = getPixel(x,y);  if (val > 0) {  val = ((val - mean) / sd);  setPixel(x,y, val);  }  }  }  }  setSlice(1);  }  //------------------------------------------------------------  //open Transmission image and process for analysis  //--------------------------------------------------------------  function TxOpen() {  open();  //run("Threshold...");  setAutoThreshold(); //perhaps better with rigorous cut-off  run("Convert to Mask", " black");  run("Divide...", "stack value=255"); // Divide all pixels by 255 to create true binary image of 0s and 1s  run("Color Balance...");  run("Enhance Contrast", "saturated=0.5"); // set color scale to 0-1  return nImages //number of opened image is most recently created  }  //---------------------------------------------------------------  // calc volume, sumSD and volume*SD in sick arm  // in: SD_cutoff - cutoff level for inclusion in "sick" volume  // out: writes to global vars - sArm_volScore, sArm_SDScore.  //---------------------------------------------------------------  function calcVolSD(SD_cutoff) {  tSum = 0;tot_pixel = 0;  for (i=hArmRoiCount; i<sArmRoiCount; i++) {  roiManager("select", i);  getSelectionBounds(xc,yc, wid, hei);  for (y=yc; y<(yc+hei); y++) {  for (x = xc; x < (xc+wid); x++) {  val = getPixel(x,y); //z already set by previous roi selection  if (val >= SD_cutoff) { //pixel value inside Roi  tSum = tSum + val; //sum all values for severity score  tot_pixel = tot_pixel +1; //sum all pixels for size score  } //end if  } //next x  } //next y  } //next i  print("Severity score: ", tSum); sArm_VolScore = tot_pixel;  print("size score (pixels): ", tot_pixel); sArm_SDScore = tSum;  }  //----------------------------------------------------------------------  // calc volume. In:total pixels. Out: returns vol  //----------------------------------------------------------------------  function calcVolume(tot_pixels) {  getVoxelSize(w, h, d, u); //wid hei depth unit  return tot_pixels*w*h*d;  } | |
|  |  |  |
